# Inpatient point-prevalence screening of New Delhi Metallo-β-lactamase (NDM)–producing Enterobacaterales and *Candida auris*

**DOI:** 10.1017/ash.2022.159

**Published:** 2022-05-16

**Authors:** Christian Greco, Heather Smith, Candice Fearon, Jennifer Flaherty, Simona Kendrick, Kimberly Malcolm, Marcy McGinnis, Manisha Shah, Kadiatu Banjoko, Anjali Zedek, Justin Smyer, Shandra Day, Nora Colburn, Christina Liscynesky, Michael Haden

## Abstract

**Background:** Carbapenem-resistant Enterobacterales (CRE) are an increasing threat to patient safety but only a small percentage of CRE identified are NDMs. Since 2018, clinical CRE isolates have been submitted to the Ohio Department of Health for sequencing and NDM cases have notably increased since that time. *Candida auris* is an emerging pathogen with similar risk factors for colonization as CRE. **Methods:** A point-prevalence study was initiated after an index patient was identified with NDM CRE infection or colonization during their inpatient admission. Two patient populations were included in the study: current patients on the same unit as the index patient and currently hospitalized patients who overlapped on any unit with the index patient for at least 72 hours. Patients had perirectal screening for CRE (via PCR) and axilla or groin screening for *C. auris* (via Xpert Carba-R Assay). Patients were excluded if they had been discharged, expired, or refused testing. **Results:** We completed 5 point-prevalence studies from March 21, 2021, to October 15, 2021. The index patients were admitted at different times and across 2 campuses including medical, cardiac, and surgical ICUs as well as medical-surgical and inpatient rehabilitation units. Moreover, 3 species of NDM were identified from urine and 2 species were identified from bronchoalveolar lavage: *Enterobacter hormaechei*, *Citrobacter freundii*, and *Enterobacter cloacae* complex. *C. freundii* and *E. cloacae* complex both had dual mechanisms of NDM and KPC. Although some of the index patients overlapped temporally within the health system, none overlapped in the same unit or building. None of the patients had recently received health care outside the United States, although 1 patient had emigrated from Togo >5 years prior and 4 had had prior local healthcare exposure within 12 months of admission. Also, 147 patients were identified for screening; 105 consented, 32 declined, and 10 were excluded due to being discharged, deceased, or unable to consent. Inpatient point-prevalence screening tests for all patients tested (n = 105) were negative for NDM CRE and *C. auris*. **Conclusions:** Despite an increase of inpatients with NDM CRE, evidence of patient-to-patient transmission was not identified, likely resulting from adherence to standard precautions. The diversity of species and lack of international travel suggests that these patients likely acquired NDM CRE from a local reservoir in the community or healthcare settings. Given the continued increase in NDM CRE without traditional risk factors, it is critical for hospitals and public health agencies to collaborate to identify these organisms and that they develop surveillance programs to clarify risk factors for colonization.

**Funding:** None

**Disclosures:** None

